# Current Understanding of Gut Microbiota in Mood Disorders: An Update of Human Studies

**DOI:** 10.3389/fgene.2019.00098

**Published:** 2019-02-19

**Authors:** Ting-Ting Huang, Jian-Bo Lai, Yan-Li Du, Yi Xu, Lie-Min Ruan, Shao-Hua Hu

**Affiliations:** ^1^Department of Psychiatry, First Affiliated Hospital, Zhejiang University School of Medicine, Hangzhou, China; ^2^The Key Laboratory of Mental Disorder’s Management of Zhejiang Province, Hangzhou, China; ^3^Brain Research Institute of Zhejiang University, Hangzhou, China; ^4^Department of Mental Health, Ningbo First Hospital, Ningbo, China

**Keywords:** gut microbiota, mood disorder, brain-gut-microbiota axis, gene sequencing techniques, human study

## Abstract

Gut microbiota plays an important role in the bidirectional communication between the gut and the central nervous system. Mounting evidence suggests that gut microbiota can influence the brain function via neuroimmune and neuroendocrine pathways as well as the nervous system. Advances in gene sequencing techniques further facilitate investigating the underlying relationship between gut microbiota and psychiatric disorders. In recent years, researchers have preliminarily explored the gut microbiota in patients with mood disorders. The current review aims to summarize the published human studies of gut microbiota in mood disorders. The findings showed that microbial diversity and taxonomic compositions were significantly changed compared with healthy individuals. Most of these findings revealed that short-chain fatty acids-producing bacterial genera were decreased, while pro-inflammatory genera and those involved in lipid metabolism were increased in patients with depressive episodes. Interestingly, the abundance of *Actinobacteria*, *Enterobacteriaceae* was increased and *Faecalibacterium* was decreased consistently in patients with either bipolar disorder or major depressive disorder. Some studies further indicated that specific bacteria were associated with clinical characteristics, inflammatory profiles, metabolic markers, and pharmacological treatment. These studies present preliminary evidence of the important role of gut microbiota in mood disorders, through the brain-gut-microbiota axis, which emerges as a promising target for disease diagnosis and therapeutic interventions in the future.

## Introduction

Mood disorders, including depressive disorder and bipolar disorder (BD), affect approximately 10% of the world’s population, causing significant individual and socioeconomic burdens (Wittchen, 2012). Compared with the general population, people with mood disorders tend to have higher rates of mortality and a decreased life expectancy (Angst et al., 1999; Kessing et al., 2015). However, the underlying mechanisms of mood disorders are not sufficiently characterized. Genetic and environmental factors contribute to the major causes of mood disorders, such as genetic vulnerability and susceptibility (Sullivan et al., 2012), chronic non-infectious inflammation (Rosenblat et al., 2014), oxidative stress (Zhao et al., 2017), neurotransmitter imbalance (Pralong et al., 2002), insufficient signaling by neurotrophic factors (Castren and Kojima, 2017), and neuroendocrine abnormalities (Linkowski, 2003). In the last decade, the potential role of gut microbiota in the pathogenesis of mood disorders has attracted considerable attention. Until now, no article has comprehensively reviewed all the human studies investigating the role of the gut microbiota in mood disorders.

The human commensal microbiota inhabits various body surfaces including the skin, nose, oral cavity, vagina, stomach, and intestine (Marsland and Gollwitzer, 2014). The human intestine contains 10 to 100 trillion microbes, which is almost 10 times greater than the total number of human cells (Bäckhed et al., 2005). The predominant bacterial phyla in the human gastrointestinal tract (GI) are *Firmicutes*, *Bacteroidetes*, *Proteobacteria*, *Actinobacteria*, *Fusobacteria*, and *Cyanobacteria* (Bäckhed et al., 2005; Marsland and Gollwitzer, 2014). Furthermore, some researchers regard the human microbiota as the second genome, which contains 100 times the number of genes of the human genome (Bäckhed et al., 2005; Grice and Segre, 2012). The normal gut ecosystem is beneficial in maintaining human health, which can be classified into metabolic, protective, structural, and histological functions (Prakash et al., 2011). The microbiota changes dynamically during individual growth (Clemente et al., 2012). However, the gut microbiota can be influenced by various factors, such as a genetic basis (Kurilshikov et al., 2017), environment (Chen et al., 2018b), mode of delivery (Dominguez-Bello et al., 2010), diet (Patman, 2015), antibiotics (Bokulich et al., 2016), and probiotics and prebiotics (Preidis and Versalovic, 2009). Dysbiosis in gut microbiota was found to be associated with many systemic disorders, such as functional bowel disorders (Mayer et al., 2014), inflammatory disease (Clemente et al., 2018), atherosclerosis (Jie et al., 2017), metabolic disease (Bouter et al., 2017), and neuropsychiatric disorders (Sharon et al., 2016). It has been reported that reduction of certain microbes that could produce short-chain fatty acids (SCFAs) was observed in inflammatory bowel disease and autoimmune diseases, and dysbiosis in gut microbiota was associated with higher levels of inflammation (Clemente et al., 2018). Furthermore, it was proven that obesity was associated with a lower ratio of *Bacteroidetes* to *Firmicutes* and the ratio increased after weight loss (Ley et al., 2006).

The alterations in the human gut microbiota composition have also been linked to a variety of neuropsychiatric disorders, including mood disorders, autism spectrum disorder (ASD), schizophrenia and Parkinson’s disease (PD) (Cenit et al., 2017). Studies indicated that altered gut bacterial communities could substantially influence the central physiology. Furthermore, many patients who suffered from GI discomfort were more likely to comorbid with mental disorders (Mussell et al., 2008; Lee et al., 2015). The GI symptoms in patients with irritable bowel syndrome (IBS) significantly improved after receiving psychotropic treatments (Palsson and Whitehead, 2002). The altered gut microbiota composition in patients with depression was related to abnormalities in hypothalamic–pituitary–adrenal (HPA) axis function, intestinal low-grade inflammation and an imbalanced neurotransmitter metabolism via the brain–gut–microbiota axis (Kelly et al., 2016). Therefore, gut microbial dysregulation may contribute to the pathogenesis of mental disorders, supporting the hypothesis of a pathological process of bidirectional communication between the gut and the brain.

The aim of this current review is thus to first introduce the brain-gut-microbiota axis, briefly describe evidence from animal studies and other neuropsychiatric disorders relevant to the brain-gut-microbiota axis, then to focus on human studies in patients with mood disorders, and lastly to discuss the cause-effect relationship between the gut dysbiosis and mood disorders. We also discuss the limitations in previous studies and propose prospective future investigations.

## The Brain-Gut-Microbiota Axis

Gut microbiota modulates brain development and function and the brain in turn interacts with gut bacteria via neuroimmune, neuroendocrine pathways, and the nervous system. This bidirectional communication system is commonly called the brain-gut-microbiota axis (Rhee et al., 2009). Through this bidirectional communication system, signals from the brain can influence the physiological effects of the gut, including motility, secretion and immune function, and messages from the gut can influence the brain function with regard to reflex regulation and mood states (O’Mahony et al., 2011). Chronic stress could affect the gut microbiota composition, which is associated with the activation of the HPA axis and an elevation in the pro-inflammatory status (Bailey et al., 2011; O’Mahony et al., 2011). The hyperactivity of the HPA axis promotes cortisol secretion and induces a pro-inflammatory response. The intestinal mucosal barrier and blood–brain barrier are important gates for substance transfer. The cortisol can increase the permeability of the intestinal tract and blood–brain barrier, thus facilitating the mutual communication between the gut microbiota and the central nervous system (CNS). In addition, the microbial composition can be interfered with by a pathogen infection or by microecological treatment (O’Toole and Cooney, 2008). The change in microbiota has a direct effect on the immune system, and the disrupted balance between pro-inflammatory and anti-inflammatory cytokines further affects brain function (Duerkop et al., 2009). The vagus nerve (VN), as a major anatomical pathway connecting the enteric nervous system (ENS) and the CNS, also plays a key role in microbiota–brain interactions (Cryan and Dinan, 2012).

### Gut Immune System

Gut microbiota is an important component of the development of a gut immune system and gut immunological homeostasis is influenced by host–microbe interactions (Furusawa et al., 2013). Germ-free (GF) mice exhibited an underdeveloped immune system and immune function, which could be restored by the colonization of certain bacteria, such as segmented filamentous bacteria (Talham et al., 1999). Symbiotic microbes maintain the immune balance through both direct and indirect pathways (Petra et al., 2015). On the one hand, microbial-associated molecular factors, including lipopolysaccharide, bacterial lipoprotein, flagellin, CpG oligodeoxynucleotide (a ligand to Toll-like receptor 9 expressed in endosomes of dendritic cells), can activate immune cells as well as toll-like receptors to promote the release of pro-inflammatory cytokines, which further increases the permeability of gut-blood barrier and blood–brain barrier, and regulates the CNS function and behavior (Petra et al., 2015; Sampson and Mazmanian, 2015). A microbiota-driven pro-inflammatory state and low-grade inflammation in dysfunctional intestinal mucosal barrier was observed in stress-related psychiatric disorders such as depression (Kelly et al., 2015). Inflammatory cytokines can also cause the over-release of the corticotropin releasing hormone, the dominant regulator of the HPA axis (de Weerth, 2017). Hyperactivity of the HPA could also contribute to increased cytokine expression in animal studies (Hueston and Deak, 2014). Indeed, hyperactivity of the HPA axis and immune activation were both observed during depressive episodes (Young, 2004; Slavich and Irwin, 2014). On the other hand, the VN, as a link between the CNS and ENS, can mediate immunoregulatory signals directly to the brain and the gut (Petra et al., 2015; Breit et al., 2018).

### The Neuroendocrine Pathway

Gut microbiota can secrete a series of neurotransmitters, such as γ-aminobutyric acid (GABA) (Barrett et al., 2012), acetylcholine (Stephenson and Rowatt, 1947), serotonin (Mittal et al., 2017), dopamine (Asano et al., 2012; Mittal et al., 2017), and histamine (Devalia et al., 1989). For instance, *Lactobacillus* spp. produces GABA and acetylcholine; *Bifidobacterium* spp. produces GABA; *Escherichia* spp. produces noradrenalin and serotonin; *Bacillus* spp. produces noradrenalin and dopamine; *Saccharomyces* spp. produces noradrenalin; *Candida* spp., *Streptococcus* spp., and *Enterococcus* spp. produces serotonin (Cryan and Dinan, 2012). Notably, More than 90% of the neurotransmitter, serotonin, in the human body is produced in the gut, which can affect emotion regulation when transmitted to the CNS (Smith, 2015). Studies in GF mice showed higher levels of noradrenalin, dopamine, and serotonin in the striatum and the hippocampus (Diaz Heijtz et al., 2011; Clarke et al., 2013). It is conceivable that neurotransmitters secreted by gut microbiota can influence the level of central neurotransmitters and then affect behavior and mood. Furthermore, bacterial metabolites, such as SCFAs (e.g., acetic acid, propionate, butyrate, isobutyric acid, valeric acid, and isovaleric acid) (Al-Lahham et al., 2010), have physiological effects including the regulation of food intake, glucose/insulin or lipid metabolism, anti-inflammatory and antitumorigenic functions, and can even activate the sympathetic nervous system (Layden et al., 2013; Borre et al., 2014; Tan et al., 2014). In addition, butyrate can alter the activity of cells located in the blood–brain barrier and exert an antidepressant-like effect in animal models (Yamawaki et al., 2012; Smith, 2015). Therefore, brain function and behavior can also be modulated by gut microbiota through the neuroendocrine pathway.

### The Neural Pathway

The communication between the gut and brain, through the neural anatomical pathway, is based on a hierarchic four-level integrative organization, including the ENS, prevertebral ganglia, the autonomic nervous system, and the CNS (Wang and Wang, 2016). Animal studies have shown that gut microbiota can activate the VN and further influence brain function and behavior (Sherwin et al., 2016). Anxiolytic and antidepressant-like behavior was observed in mice treated with *Lactobacillus rhamnosus*, but not in vagotomized mice (Bravo et al., 2011). Similar findings were reported in rats with probiotic administration of *Bifidobacterium longum* (Bested et al., 2013). It seems that the effects of gut microbiota on the brain function are dependent on vagal activation. Furthermore, activation of the VN inhibits cytokine production, manifesting as an anti-inflammatory response (Johnston and Webster, 2009).

### Evidence From Animal Studies

Currently, evidence of the brain-gut-microbiota axis are mostly obtained from animal studies. These animal studies have verified the role of gut microbiota on modulating gut–brain interactions, through various strategies such as GF animal observation, fecal microbial transplantation, and probiotic treatment (Mayer et al., 2015). GF mice exhibited an enhanced HPA response from stress and reduced the expression of the brain-derived neurotrophic factor (BDNF) in the cortex and hippocampus, compared with specific-pathogen-free mice (Sudo et al., 2004). Moreover, the exaggerated HPA stress response in the GF mice could partially be reversed by orally inoculated *Bifidobacterium infantis* (Sudo et al., 2004). It is however worth noting that some studies showed a reduction of anxiety-like behaviors in GF mice (Neufeld et al., 2011; Clarke et al., 2013; Arentsen et al., 2015). GF mice displayed alterations in behavior and stress responses, changes in neurotransmitter levels and immune activation (Campos et al., 2016). Anxiety-like behaviors in GF mice can be normalized following the restoration of intestinal microbiota (Clarke et al., 2013). Interestingly, fecal microbiota transplantation in GF mice from depressed patients led to depression-like behaviors (Zheng et al., 2016). Furthermore, depression-like behaviors in a rat model could also be reversed with probiotic treatment (Desbonnet et al., 2008, 2010). These results suggest that the composition in gut microbiota may contribute to regulating mood and behavior, but the detailed molecular mechanisms and cause-effect relationship between gut microbiota and phenotypes of moods and behaviors are not fully understood.

## Gut Microbiota and Neuropsychiatric Disorders

Previous human studies have investigated the link between gut microbiota and a series of neuropsychiatric disorders. Alternations in gut microbial composition have been observed in children with ASD, with an increase in the *Firmicutes*/*Bacteroidetes* ratio (Williams et al., 2011; Tomova et al., 2015; Strati et al., 2017). A recent study with 35 ASD children and six healthy controls also found consistent results, and the functional analysis of this study demonstrated that butyrate/lactate-producing bacteria were decreased in ASD children (Zhang et al., 2018). Patients with schizophrenia also showed dysbiosis of gut microbiota, with a higher *Proteobacteria* abundance compared to the healthy controls (Shen et al., 2018). Another study reported an increased abundance of *Lactobacillus* in patients with first-episode psychosis, and patients with stronger microbial differences compared to the controls, showed worse treatment outcomes (Schwarz et al., 2018). It has also been proven that some neurodegenerative diseases are associated with gut microbiota. Increasing evidence shows gut microbiota changes in PD patients. Higher microbial diversity was found in Chinese and American PD patients, but not in Finnish and German patients, which may be related to regional variation (Keshavarzian et al., 2015; Scheperjans et al., 2015; Hopfner et al., 2017; Qian et al., 2018). A metagenomic shotgun analyses performed in PD patients showed that PD can potentially be identified from healthy controls with gut microbiota-based biomarkers (Bedarf et al., 2017). Alternation in gut microbial metabolites, including β-glucuronate and tryptophan metabolism, was further observed in PD patients (Bedarf et al., 2017). In addition to the diseases mentioned above, preliminary studies characterizing the gut microbiota in patients with mood disorders have also emerged recently, providing rudimentary knowledge in this field. This review will hereinafter focus on studies carried out in patients with major depressive disorder (MDD) and BD.

## Human Studies of the Gut Microbiota in Mood Disorders

**Figure 1:**
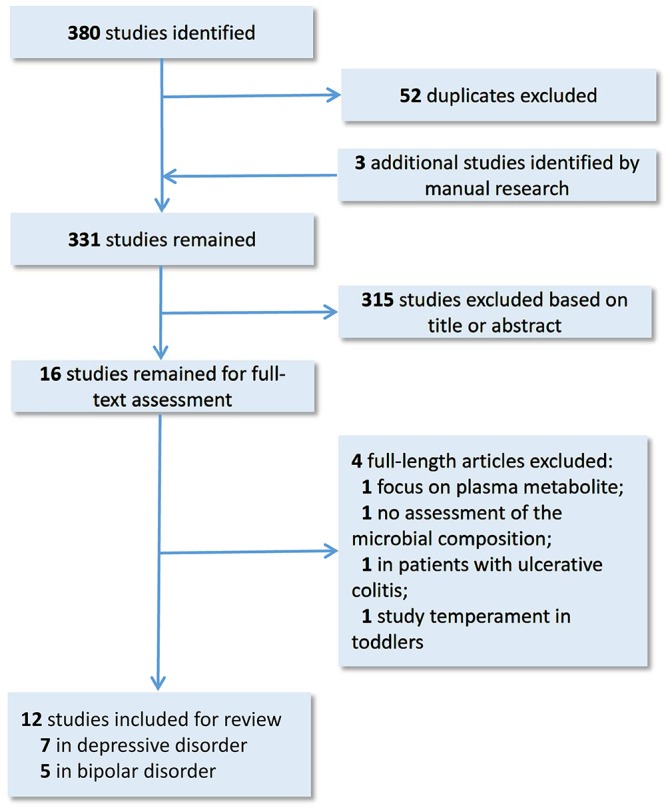
Dynamic flow chart for study selection.

The search strategy we used was in accordance with the Preferred Reporting Items for Systematic Reviews and Meta-analyses (PRISMA). We selected relevant studies before October 1, 2018, by searching PubMed, Embase and PsycINFO databases. The search keyword string used was (mood disorder OR bipolar disorder OR mania OR depression OR depressive disorder) AND gut microbiota. The results were further filtered by human studies. No language restrictions were required. We also searched the reference lists of key articles, manually. Studies eligible for inclusion needed to have investigated the characteristics of gut microbiota in patients with MDD or BD, using a high-throughput sequencing or proteomics approach. The studies needed to be full-text research articles, rather than reviews, letters, case reports, or meeting abstracts. All studies identified were screened by their titles and abstracts, as well as the full article if needed. The study selection process is shown in Figure 1. Finally, 12 research articles, on the gut microbiota in mood disorders, were included for further review. Overall, seven studies were carried out on MDD, and five studies on BD. 16S rRNA gene sequencing is the currently mainstream tool to identify phylogenetic relationships between various bacteria. The 16S rRNA gene exists in all bacteria. Its function remains conservative over time, and it is large enough to distinguish between different bacteria (Patel, 2001). These studies further conducted a correlation analysis to explore the relationships between the gut microbial features and demographic, immune, metabolic and clinical data in MDD and BD patients. The sample size across studies ranged from 10 to 58 in MDD subjects, and 31 to 115 in BD subjects. Among MDD and BD patients, the mean age was 39.5 and 39.9; the mean female ratio was 45.7 and 55.8%; and the mean body mass index (BMI) was 23.1 and 26.3, respectively. Details of these studies are provided in Table 1. We will discuss the main findings in MDD and BD separately below.

**Table 1:** Human studies of the gut microbiome in mood disorders.

Disease	Publication	Sample size	Mean age	Gender (female ratio %)	Alpha diversity	Taxonomic differences
MDD	Naseribafrouei et al., 2014	MDD: 37	MDD: 49.2	MDD: 54.1	No difference	MDD: *Lachnospiraceae* ↓; *Bacteroidales*, *Alistipes*, and *Oscillibacter*↑
		HCs: 18	HCs: 46.1	HCs: 61.1		
	Jiang et al., 2015	MDD: 46	MDD: 26.2	MDD: 41.3	A-MDD:↑	MDD: *Bacteroidetes*, *Proteobacteria* and *Actinobacteria*↑; *Firmicutes*↓
		(A-MDD:R-MDD = 29:17)	(A-MDD:R-MDD = 25.3: 27.1)	(A-MDD:R-MDD = 38: 47)	no difference in R-MDD	*Enterobacteriaceae* and *Alistipes*↑; *Faecalibacterium*↓
		HCs: 30	HCs: 26.8	HCs: 50		
	Aizawa et al., 2016	MDD: 43	MDD: 39.4	MDD: 41.9	No mentioned	MDD: *Bifidobacterium*↓
		HCs: 57	HCs: 42.8	HCs: 61.4		Lactobacillus↓
	Zheng et al., 2016	MDD: 58	MDD: 40.6	MDD: 37.9	No difference	MDD: *Actinobacteria*↑; *Bacteroidetes*↓
		HCs: 63	HCs: 41.8	HCs: 36.5		
	Lin et al., 2017	MDD: 10	MDD: 36.2	MDD: 40	No mentioned	MDD: *Firmicutes*↑, *Prevotella*, *Klebsiella*, *Streptococcus*, and *Clostridium* XI↑; *Bacteroidetes*↓
		HCs: 10	HCs: 38.1	HHCs: 40		
	Chen et al., 2018a	F-MDD:HCs = 24:24	F-MDD:HCs = 41.5:44.0	/	No mentioned	F-MDD: *Actinobacteria*↑;
		M-MDD:HCs = 20:20	M-MDD:HCs = 40.4:42.8			M-MDD: *Bacteroidetes*↓
	Chen et al., 2018c	MDD: 10	MDD: 43.9	MDD: 50	No mentioned	MDD: *Proteobacteria* and *Bacteroidetes*↓; *Actinobacteria* and *Firmicutes*↑
		HCs: 10	HCs: 39.6	HCs: 50		
BD						
	Evans et al., 2017	BD:115 HCs: 64	BD: 50.2	BD: 83	No mentioned	BD: *Faecalibacterium* and an unclassified member from the *Ruminococcaceae* family↓
		HCs: 48.6	HCs: 40			
	Flowers et al., 2017	AAP-treated: 49	AAP-treated: 46	AAP-treated: 34	AAP-treated females:↓	AAP-treated BD patients: *Lachnospiraceae*↑ Non-AAP-treated patients: *Akkermansia* and *Sutterella* ↓
		Non-AAP-treated: 68	Non-AAP-treated: 51.7	Non-AAP-treated: 48		
	Coello et al., 2018	BD: 113 HCs: 77	BD: 31	BD: 62.5	No mentioned	BD: *Flavonifractor* ↑
		HCs: 29	HCs: 61			
	Painold et al., 2018	BD: 32 HCs: 10	BD: 41.3 HCs: 31.4	BD: 43.8 HCs: 60	Negatively correlated with illness duration	BD: *Actinobacteria* and *Coriobacteria*↑*Ruminococcaceae* and *Faecalibacterium*↓
	Guo et al., 2018	BD: 31	BD: 25.1	BD: 41.9	BD:↑, especially in BDM group	BD: *Proteobacteria*, *Ruminococcus*, *Veillonella*, and *Lanchnospira*↑; *Bacteroides* ↓
		(BDD:BDM = 12:19) HCs: 28	(BDD:BDM = 23.2: 25.4)	(BDD:BDM = 41.7: 42.1)		BDM: *Enterobacteriaceae*, *Ruminococcus*, *Megamonas*, and *Bifidobacterium adolescentis* ↑; *Bacteroides* ↓
			HCs: 27.1	HCs: 42.9		BDD: *Selenomonadales*, *Lachnospira*, *Eubacrerium*, and *Plebeius*↑

*MDD, major depressive disorder; A-MDD, active-MDD; R-MDD, responded-MDD; F-MDD, female-MDD; M-MDD, male-MDD; BD, bipolar disorder; BDD, BD patients with depressive episode; BDM, BD patients with manic episode; HCs, healthy controls; AAP-treated, atypical antipsychotic-treated*.

### Gut Microbiota and Major Depressive Disorder

To date, seven documented studies have investigated the association between MDD and gut microbiota in humans. Among these studies, four performed a microbial diversity analysis. Jiang et al. (2015) reported greater diversity of gut microbiota in MDD patients when compared with healthy individuals. While the other three studies failed to find significant differences in microbial diversity (Naseribafrouei et al., 2014; Zheng et al., 2016; Chen et al., 2018a). High microbial diversity is potentially beneficial to health, but it could easily be influenced by age, diet, and other factors (Yatsunenko et al., 2012). Additionally, all seven studies analyzed microbial composition changes in MDD patients. Naseribafrouei et al. (2014) first compared the gut microbiota in 37 depressed patients and 18 healthy controls, in whom a higher abundance of order *Bacteroidales*, genus *Oscillibacter* and *Alistipes*, and a lower abundance of family *Lachnospiraceae* was associated with depression. Another study investigated the gut microbiota in active-MDD (A-MDD) patients, responded-MDD (R-MDD) and healthy controls (Jiang et al., 2015). In this study, the *Proteobacteria*, *Bacteroidetes*, *Actinobacteria* abundance was increased, while *Firmicutes* was decreased in both A-MDD and R-MDD patients compared to the healthy controls. However, higher microbial diversity was only found in A-MDD patients, not in R-MDD patients. At lower taxonomic levels, increased *Enterobacteriaceae* and *Alistipes* and decreased *Faecalibacterium* were observed in MDD patients. Furthermore, the *Faecalibacterium* genus was negatively associated with the severity of depressive symptoms (Jiang et al., 2015), whereas *Prevotella* and *Klebsiella* were found positively correlated with the depression score in another study (Lin et al., 2017). In addition, this study reported more phylum *Firmicutes*, genus *Prevotella*, *Klebsiella*, *Streptococcus*, and *Clostridium* XI and less *Bacteroidetes* in depressed patients (Lin et al., 2017). Gut beneficial bacteria, such as *Bifidobacterium* and *Lactobacillus*, were also reduced in MDD patients (Aizawa et al., 2016). Zheng et al. (2016) found increased *Actinobacteria* and *Bacteroidetes* abundance and reduced *Firmicutes* in MDD patients, consistent with Jiang’s finding. However, Chen et al. (2018c) found that *Firmicutes* and *Actinobacteria* were increased, whereas *Bacteroidetes* and *Proteobacteria* were reduced in 10 MDD patients compared to 10 healthy controls. Abundance of *Faecalibacterium* was also found to be associated with depression severity (Chen et al., 2018c). Proteomics analysis further indicated the disordered bacterial proteins involved in carbohydrate and amino acid metabolism (Chen et al., 2018c). Only one study explored the sex differences of gut microbiota in MDD patients, demonstrating that levels of *Actinobacteria* phylum was increased in females and the *Bacteroidia* class was decreased in males (Chen et al., 2018a).

From the current findings in MDD patients, we found increased levels of phylum *Actinobacteria*, order *Bacteroidales*, family *Enterobacteriaceae*, genus *Alistipes* and deceased family *Lachnospiraceae*, genus *Faecalibacterium* were associated with depression in most studies. However, the change of *Bacteroidetes* was not consistent among these studies. Phylum *Actinobacteria* is involved in lipid metabolism (Painold et al., 2018), thus indicating that more *Actinobacteria* in depressed patients were possibly related to dyslipidemia. *Bacteroidetes* and *Bacteroidales* were found to be associated with complex polysaccharide hydrolysis, and low *Bacteroidetes* and *Bacteroidales* levels were shown to be associated with metabolic diseases, such as obesity and diabetes (Ley et al., 2006; Zhang et al., 2013). The *Enterobacteriaceae* family is a natural inhabitant of the intestinal tract, and the inflammatory status in gut microbiota is particularly beneficial for the proliferation of *Enterobacteriaceae* (Zeng et al., 2017). *Alistipes* genus was associated with triggering inflammation and tumorigenesis in an IL-6-dependent manner (Moschen et al., 2016). A study on BALB/c mice showed a significant increase in *Alistipes* abundance when exposed to stress (Bangsgaard Bendtsen et al., 2012). Recent studies have shown that *Alistipes* was associated with a higher risk of obesity (Kang et al., 2018), IBS (Saulnier et al., 2011), and immune deficiency syndrome (McHardy et al., 2013). Notably, *Faecalibacterium* and *Lachnospiraceae* were important for the biosynthesis of the microbial product butyrate, which has anti-inflammatory effects, partly due to the decrease of pro-inflammatory cytokine synthesis and the increase of anti-inflammatory cytokine secretion (Duncan et al., 2007; Sokol et al., 2008). Consistent findings showed a negative association between *Faecalibacterium* and depressive symptoms, but the underlying mechanisms connecting *Faecalibacterium* with depression remain unclear.

### Gut Microbiota and Bipolar Disorder

To date, the association between BD and gut microbiota has been documented in only five studies. Evans et al. (2017) first investigated the gut microbiota characteristics in 115 BD patients compared to 64 healthy controls. The levels of *Faecalibacterium* and a member of the *Ruminococcaceae* family of *Firmicutes* phylum were decreased, and the *Faecalibacterium* abundance was negatively correlated with self-reported symptoms and depressive severity (Evans et al., 2017). This finding is consistent with the study by Painold et al. (2018). The latter also reported higher levels of phylum *Actinobacteria* and class *Coriobacteria* in BD patients (Painold et al., 2018). Based on the severity of depressive symptoms, the *Clostridiaceae* family and *Roseburia* genus were more abundant in healthier BD patients, while the *Enterobacteriaceae* family was more abundant in clinically depressive patients (Painold et al., 2018). In addition, microbial diversity was negatively correlated with illness duration (Painold et al., 2018). Inflammatory and metabolic profiles, such as serum IL-6, lipids, tryptophan, BMI, were associated with specific bacteria in BD patients, and were all positively correlated with the genus *Lactobacillus* abundance (Painold et al., 2018). Another study found higher microbial diversity in BD patients compared to healthy subjects, especially in patients with manic episodes (Guo et al., 2018). In patients with different mood statuses, the relative abundance of *Escherichia coli* and *Bifidobacterium adolescentis* was higher in manic individuals, while *Stercoris* was higher in depressed individuals (Guo et al., 2018). *Flavonifractor* genus was also identified to be associated with BD patients, especially female patients who in smoke (Coello et al., 2018). However, no difference of gut microbiota was observed between unaffected first-degree relatives of BD patients and healthy subjects (Coello et al., 2018). To date, only one study has explored the influence of atypical antipsychotic (AAP) treatment on gut microbiota in BD patients. Significantly decreased microbial diversity was revealed in AAP-treated females (Flowers et al., 2017). Furthermore, AAP treatment was associated with an increased abundance of *Lachnospiraceae* and a decreased abundance of *Akkermansia* and *Sutterella*.

As shown in these studies, the gut microbiota in BD patients tend to harbor higher phylum *Actinobacteria*, order *Coriobacteriales*, family *Coriobacteriaceae, Enterobacteriaceae*, genus *Flavonifractor* and lower genus *Faecalibacterium*, and the abundance of *Bacteroides*. *Actinobacteria*, *Coriobacteriales*, *Coriobacteriaceae*, and *Bacteroides* was proven to be associated with lipid and glucose metabolism. Therefore, the increased risk for metabolic disturbance in BD patients may be related to the altered abundance of these bacteria (McElroy and Keck, 2014). *Flavonifractor* abundance was increased in BD patients, and may be correlated to the influence of oxidative stress and inflammatory reactions (Coello et al., 2018). *Faecalibacterium*, which was also decreased in MDD patients, was shown to be beneficial for human health through anti-inflammatory activities in the gut. As a whole, the disordered gut microbial communities in BD patients are linked to an abnormal inflammatory, metabolic process and oxidative stress, as well as the disease itself.

### Gut Microbiota Difference Between Major Depressive Disorder and Bipolar Disorder

**Figure 2:**
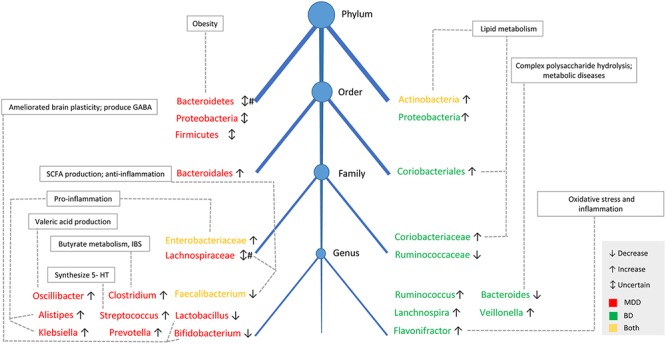
The taxonomic tree and function of main fecal bacterial clades in MDD and BD. The taxonomic tree shows the common and different features of gut microbiota composition between MDD and BD patients, as well as the physiological functions of the specific bacteria. Decreased in SCFAs-producing genera and increased in pro-inflammatory genera was reported both in patients with BD and MDD. # There were four studies showing lower *Bacteroidetes* levels in MDD patients, with one study showing higher abundance of *Bacteroidetes* in MDD patients. Similarly, the abundance of *Lachnospiraceae* was decreased in two studies in MDD patients, but another study showed *Lachnospiraceae* was increased in MDD patients. SCFAs, short-chain fatty acids; IBS, irritable bowel syndrome; 5-HT, 5-hydroxytryptamine.

Major depressive disorder and BD are two different psychiatric disorders according to the DSM-5, with different clinical symptoms, therapies, and prognosis. The different gut microbial characteristics in BD and MDD patients may indicate different etiologies of these two diseases. However, some findings were similar in BD and MDD patients. For example, a higher abundance of *Actinobacteria*, *Enterobacteriaceae* and lower *Faecalibacterium* was reported in both diseases, and these bacteria were related to lipid metabolism and an inflammatory response, which may contribute to the disturbance in lipid metabolism and pro-inflammatory activities in affected patients (Sowa-Kucma et al., 2018). In addition, other different findings in the gut bacteria of BD and MDD patients should be mentioned. The SCFA-producing bacterium, *Lachnospiraceae* family, was decreased in MDD patients in most studies, but *Lachnospira* genus was increased in BD patients. Pro-inflammatory genera, *Alistipes* and *Klebsiella*, were increased in MDD patients, but not reported in BD patients. Furthermore, bacteria communities identified in MDD patients were involved more in serotonin, GABA, valeric acid and butyrate production metabolism, but were more likely to be associates with lipid metabolism in BD patients. Details are shown in Figure 2.

### The Impact of Psychotropic Medication on the Gut Microbiota

What impact do psychotropic medications have on the human gut microbiota? So far, few studies have addressed this issue in humans. Among the studies included for review, only one has described the changes in microbiota from AAP treatment in BD patients. In this study, APP-treated patients had a higher BMI compared to non-treated patients, and the microbial diversity was decreased only in female treated patients, but not in male treated patients (Flowers et al., 2017). In addition, treated patients without obesity, showed significantly decreased *Akkermansia* (Flowers et al., 2017). This bacterium was reported to present an inverse association with inflammation, insulin resistance, and lipid metabolism (Schneeberger et al., 2015). Therefore, its reduction in BD may predispose patients to inflammatory conditions and metabolic perturbations. In patients with schizophrenia, chronic treatment with risperidone was associated with weight gain and a lower ratio of *Bacteroidetes* to *Firmicutes* (Bahr et al., 2015). Interestingly, chemically different antipsychotics can exert inhibitory effects on the growth of gut-originated microbial strains, indicating that these non-antibiotics have antibiotic-like side effects (Maier et al., 2018). In addition to antipsychotics, some antidepressants are also considered to have antimicrobial effects (Macedo et al., 2017). Some other possible mechanisms should be taken into consideration. Most psychotropic medications target neurotransmitters and their receptors, including serotonin, dopamine and noradrenalin, which can also be produced by gut microbiota and can potentially have feedback on the bacteria. Furthermore, improvement of clinical symptoms, through psychotropic medications, may also influence the diversity and composition of gut microbiota. The gastrointestinal side effects of these drugs, such as constipation and diarrhea, may also affect the commensal bacteria. Although the underpinnings are not fully understood, alterations of gut microbiota in relation to psychotropic drugs, also seem to contribute to weight gain, metabolic disturbance and inflammatory activities in patients. The drug–microbiota interactions provide promising paths to understanding and controlling their off-target side effects.

## Discussion

Accumulating evidence on the role of the brain-gut-microbiota in neuropsychiatric diseases has emerged in recent years. Herein, we focus on the human studies of gut microbiota pertaining to mood disorders. Compared to healthy individuals, MDD and BD patients showed significant changes in gut microbial diversity and composition. In depressed patients, decreased microbial diversity was found in most studies. According to different studies, a consistent increase in the abundance of *Actinobacteria*, *Enterobacteriaceae* and a decrease in *Faecalibacterium* was revealed. These findings indicate that decreased SCFAs-producing genera and increased pro-inflammatory genera may be related to chronic, low-grade systemic inflammation in patients with mood disorders. Furthermore, specific gut bacteria were also associated with inflammatory markers and metabolic profiles, disease severity, duration of illness, psychiatric symptoms, and pharmacological treatment.

Current studies shed a light on the potential of using gut microbial markers to distinguish between patients with mood disorders from unaffected healthy individuals. However, these studies were all cross-sectional, and the cause-effect relationship between the mood disorder and gut microbiota remains unclear. Among the studies included, only two investigated the microbiota characteristics between active depressed patients and responder patients (Jiang et al., 2015; Painold et al., 2018). The gut microbiota composition in responder patients also showed a significant difference in healthy controls, indicating that the improvement of clinical symptoms could not restore the human gut microbiota to nearly the same state as the gut microbiota of healthy individuals. Longitudinal studies, with the confounding factors controlled and patients in different statuses (depression, mania, and remission), are needed to clarify the causal relationship between gut microbiota and mood disorders.

There are some major limitations in the current studies of human gut microbiota in mood disorders. Most studies included a small size sample of subjects, which would inevitably weaken the study stringency. Consistent demographic and clinical characteristics of recruited subjects are needed. However, the gut is a complicated ecosystem, which can be influenced by various factors, such as age (O’Toole and Jeffery, 2015), genetics (Bonder et al., 2016), diet (David et al., 2014), and regional variations (He et al., 2018). Recruited subjects in most studies did not receive a standardized diet, and the geographical effect was also not strictly controlled. Furthermore, all studies were cross-sectional, without evaluating influences of antipsychotic medications on the gut microbiota. Although some studies have evaluated the associations between the depression severity and gut microbiota, other domains, such as the relationship of gut microbiota with psychotic symptoms, cognitive function and sleep disturbances were not further investigated. In addition, the disease status of patients should be clearly classified. Patients with different mood statuses may have distinct gut microbial composition.

## Conclusion

Current research on gut microbiota and mood disorders is still at its early stage. Growing evidence shows changed gut microbiota in patients with mood disorders, which may play an important role in disease pathology. The cause-effect relationship is still inconclusive. Future well-designed studies with new techniques, such as proteomics, metabonomics and metagenomics, are warranted to address this issue.

## Author Contributions

S-HH, L-MR, and YX contributed to the study concept and design. T-TH, J-BL, and Y-LD wrote and revised the manuscript. All authors read and approved the final manuscript.

## Conflict of Interest Statement

The authors declare that the research was conducted in the absence of any commercial or financial relationships that could be construed as a potential conflict of interest.
